# Policy on Hazard Analysis and Critical Control Point (HACCP) and adherence to food preparation guidelines: a cross sectional survey of stakeholders in food service in Kumasi, Ghana

**DOI:** 10.1186/1756-0500-6-442

**Published:** 2013-11-04

**Authors:** Peter Agyei-Baffour, Kofi Boateng Sekyere, Ernestine Akosua Addy

**Affiliations:** 1 Department of Community Health, School of Medical Sciences, College of Health Sciences, Kwame Nkrumah University of Science and Technology (KNUST), Kumasi, Ghana; 2 Komfo Anokye Teaching Hospital (KATH), Kumasi, Ghana

**Keywords:** Hazard analysis and critical control point, Food safety guidelines, Food preparation, Ghana

## Abstract

**Background:**

Food borne diseases claim more lives and are growing public health concerns. Simple preventive techniques such as adoption and adherence to hazard analysis and critical control point (HACCP) policy can significantly reduce this disease burden. Though food screening and inspection are done, the ultimate regulation, Hazard Analysis and Critical Control Point, which is known and accepted worldwide, appears not to be popular among food operators in Ghana. This paper examines the level of awareness of the existence of policy on hazard analysis and critical control point (HACCP) and its adherence to food preparation guidelines among food service providers in Ghana.

**Results:**

The results revealed the mean age of food providers as 33.1 years with a standard deviation of 7.5, range of 18–55 years, more females, in full time employment and with basic education. Of the fifty institutional managers, 42 (84%) were senior officers and had worked for more than five years. Education and type of food operator had strong statistically significant relationship with the implementation of HCCP policy and adherence with food preparation guidelines. The enforcement of HACCP policy and adherence with food safety guidelines was led by the Ghana Tourist Board, Public Health officers, and KMA, respectively. While a majority of food operators 373**/**450 **(**83.3%) did not know HACCP policy is part of food safety guidelines, staff of food safety law enforcement 44/50 (88%) confirmed knowing that food operators were not aware of the HACCP policy.

**Conclusion:**

The study documents evidence on the practice of food safety principles or HACCP policy or adherence to food preparation guidelines. Existing food safety guidelines incorporate varying principles of HACCP, however, awareness is low among food operators. The implication is that food production is likely to fall short of acceptable standards and not be wholesome putting consumers at health risk. Repeating this study in rural and urban areas in Ghana is necessary to provide much more evidence to inform food safety guidelines. Further studies on chemical analysis of food and implementing training modules on HACCP policy for food producers and law enforcement agencies may be helpful to improve existing situation.

## Background

The burden of food borne diseases though preventable remains huge contributing to worldwide morbidity. The WHO estimates that each year, unsafe food makes at least two billion people, representing about one third of the global population ill worldwide. Simple prevention techniques could significantly reduce this burden of diseases which can cause serious illness or death [1-3]. Food borne diseases claim more lives and is reported very often in health facilities. Foodborne diseases are widespread and growing public health problem, both in developed and developing countries [4]. The global incidence of food borne diseases is difficult to estimate, but it has been reported that in 2005 alone, 1.8 million people died from diarrhoea and related diseases [5]. A greater proportion of these cases can be attributed to contamination of food and drinking water. In industrialized countries, the percentage of the population suffering from food borne diseases each year has been reported to be up to 30%. For instance, in the United States of America (U.S.A), around 76 million cases of food borne diseases, resulting in 32,500 hospitalizations and 50,000 deaths are estimated to occur each year [6,7].

While less well documented, developing countries bear the brunt of the problem due to the presence of a wide range of food borne diseases, including those caused by parasites. The high prevalence of diarrhoea diseases in many developing countries affirms the food safety problems. Though most food borne diseases are sporadic and often not reported, food borne disease outbreaks may take on massive proportions of the population risk. For example, in 1994, an outbreak of Salmonellosis due to contaminated ice cream in the U.S.A affected an estimated 22,400 persons [8]. Similarly, in 1988, an outbreak of hepatitis A, resulting from the consumption of contaminated clams affected some 30,000 individuals [9,10]. In Zimbabwe, cholera epidemic claimed over 1,500 lives in August, 2008. Additional 29,131 suspected cases were reported. Efforts at reducing food borne disease are under way and they include enforcement of laws on food safety, health education and more importantly, the use of hazard analysis and critical control point (HACCP) principles [11,12]. In Ghana food borne diseases such as cholera and typhoid fever are among the top ten diseases in most health facilities. However, there is little evidence on policy and adherence to food guidelines among stakeholders of the food industry in Ghana. The objective of the study is to assess whether food service providers in Ghana are aware of existing policy on hazard analysis and critical control point (HACCP) and the extent to which they adhere to food preparation guidelines.

### Situating the study

The study was situated within the context of HACCP. HACCP is a management system in which food safety is addressed through the analysis and control of biological, chemical, and physical hazards from raw material production, procurement and handling, to manufacturing, distribution and consumption of the finished product [13,14]. HACCP is designed for use in all segment of food industry from growing, harvesting, processing, manufacturing, distributing and merchandising to preparing food for consumption. Prerequisites programmes such as Current Good Manufacturing Practices (CGMPS) are essential foundation for the development and implementation of successful HACCP plans. HACCP is based on seven principles; conducting a hazard analysis, determining the critical control points (CCPs), establishing critical limits, monitoring procedures, corrective actions, verifying procedures, establishing record-keeping and documentation procedures [15]. Food safety system based on these principles of HACCP have been applied in food processing plants, retail food stores and food services operations [16]. HACCP have been universally accepted by government agencies, trade associations and the food industry around the world [17,18] as effective tool to ensure food safety. The production of safe food products require that the HACCP system is built upon a solid foundation of prerequisite programmes. Each segment of the food industry must provide the condition necessary to protect food while it is under their control. HACCP is a systematic approach to the identification, evaluation, and control of food safety hazards based on these seven principles [19].

It is important to note that, the success of a HACCP system depends on the type of organization [20], educating and training management and employees on the importance of their role in producing safe foods. This also includes information on the control of food borne hazards at the all stages of the food chain. It is important to recognize that employees, first understand what HACCP is and then learn the skills necessary to make it function properly. Also, specific training on working instructions and procedures that outline the tasks of employees in monitoring each aspect of HACCP should be organized for people involved in food preparation. Management of food industry must provide adequate time for thorough education and training and provide personnel with materials and equipment necessary to perform these tasks. Effective training is an important prerequisite to successful implementation of a HACCP [21,22].

In Ghana, there is little information on the use of HACCP and adherence to food guidelines among food operators. The Food and Drug Authority, Ghana, is the main organization which is responsible for ensuring that food produced and sold to the public is wholesome. It works with the districts assemblies through the Public Health Units or Environmental Health Departments to ensure that through the District Assembly Bye Laws, the Food and Drugs Act and the criminal code 1960 section 286 are strictly adhered to. A cursory review of literature on food policy and HACCP suggested rather that, no mention is made of the HACCP policy in any of the laws or guidelines. The Ghana Food and Drugs Law and PNDC Law 305, also failed to present information regarding implementation and certification of HACCP. Hence there is nothing documented on the application and certification on HACCP in any of the policies in Ghana. The only organizations that are reported to use HACCP are the Pharmaceutical companies and Guinness Ghana Ltd probably because of their strong relationship with the international standard organizations. This shows that there is paucity in knowledge and implementation and certification of HACCP [23].

### Food safety policies

Safe food is important in preventing food borne diseases. Merican [24], has explained that, it is a legal requirement to document food preparation procedures to ensure that food served is safe to eat. Food handlers must read the food safety policy and sign to show that they understand its content. A supervisor must check monthly that the food safety policy is being adhered to and record outcome in the food safety diary. The need to protect the public against infections is of paramount importance in the food industry. Food safety policies and procedures are therefore used to create safety management and such safety polices include procedures, quality assurance and the use of HACCP.

Ensuring food safety is a transdisciplinary task involving, government ministries, departments and agencies (MDAs). While the enactments are made by Parliament and the regulations made pursuant to these enactments provide the main corpus of food law, the work of these MDAs are critical for the successful development and application of food laws and improvement in food safety [25]. Harmonization of the activities in these MDAs is important in ensuring food safety. The status of food safety legislation institutions are set up with specific mandates derived from various legislative instruments. Most of these legislations need urgent revision to align with the current trends in modern food regulations. There are two areas of concern; Separation of standards setting and advice responsibilities from standard control and enforcement as well as separation of risk assessment and advice from risk management. Unfortunately, low compliance with food safety policies [26], and factors influencing compliance to food safety guidelines have been described as complicated [27] and include education and strong institutions [28,29].

In Ghana, the Ministry of Health, Standard Board and Food and Drug Board oversee issues about food safety. For instance, the major aims of Ghana Standard Board (GSB) includes; establishment and promulgation of standards with the object of ensuring high quality in goods produced in Ghana, whether for local or for export; and promoting standardisation in industry and commerce; promoting industrial efficiency and development, promoting standards in public and industrial welfare, health and safety. Again, one of the core mandates of the GSB includes; Article 3(2) (d) to maintain the necessary machinery to ensure that goods prepared and manufactured for export are distinctly marked for export only, and to provide for issue of a certificate to the effect that goods comply with known requirement of standards in the country to which they are or about to be consigned, before the export of such goods are permitted. Others include; Article 3(2) (k) to cooperate with representatives of any industry, or with any government department, local authority, or other public bodies or persons with a view to securing the adoption of standards safety [30,31]. This is important for private sector-led standards development and application. In spite of these laudable provisions, there is little information on the practice of HACCP or adherence to food preparation guidelines as explained earlier. Undoubtedly, this is a policy input gap which needs to be addressed.

In order to contributing to filling this knowledge gap, this study was designed to generate preliminary data on the implementation of HACCP policy in food production in the study area. The data is expected to form the basis for future cross sectional studies and development of training modules to train stakeholders in food service on HACCP policy in Ghana and beyond to improve the current situation.

## Methods

### Setting and sample

A descriptive exploratory study was conducted to determine the awareness and adherence to a food safety policy (HACCP) among selected stakeholders in food service in Kumasi Metropolitan Area, Ghana, from May to October 2009. The study involved the use of structured questionnaire administered face-to-face to respondents. The study population consisted of food services staff; matrons, chefs, cooks, servers, managers of hotels, restaurants, selected food vendors and “chop bar” (local restaurants) operators. In addition, stakeholders in the food services industry who enforce laws or regulations on food safety, educate and train food services staff such as staff of Metropolitan Assembly (Environmental Health Department), Food and Drugs Board, Tourist Board and Health services staff, were part of the study. Study participants were made to sign informed consent before taking part in the study.

Kumasi was chosen for the study because of its location advantage; it is located almost at the centre of Ghana and is considered the economic nerve and thus almost all the population characteristics and economic activities in Ghana are represented. It is located in the transitional forest zone and is about 270 km north of the national capital, Accra [32].

According to the Environmental Health Department of Kumasi Metropolitan Area, Kumasi, 55% of food services staff was estimated to have been registered and medically screened and declared fit. Assuming the population is normally distributed at 95% confidence interval level and with a margin of error of 0.05, the sample size for the study was estimated from the equation, n = Z^2^ p (1-p)/d^2^; where n is sample size, d = the error margin and p, the proportion of estimated registered or screened food service staff [33], n = (1.96)^2^ [(0.37) (0.67)/(0.05)^2^, n = 442. Additionally, 50 key stakeholders (10 from each of the five institutions mentioned earlier) were selected purposively and added to the sample size; totaling 492 rounded up to 500 to account for sampling variability.

A cluster sampling was used to select respondents. The clusters were selected with probability proportion to the list of population of food vendors in each sub-metropolitan area of Kumasi. Respondents from the food vendors were systematically sampled; eligible food vendors were sampled in 5 clusters based on the regional definition of 90 food vendors per cluster. The essence of this strategy was to avoid redundancy, improve distribution of sample and minimize design effect. For the purpose of this study, a food vendor was defined as a group of people who sell or prepare food to the population.

The 50 managers otherwise known as key stakeholders were purposively selected from institutions which had been implementing food safety management system for more than ten years and were concerned about the many food scandals frequently happening all over the country and were aware of the consumers’ demands with respect to safe and quality foods. The institutions included Environmental Health Department (Kumasi Metropolitan Assembly), Food and Drugs Board (FDB), Ministry of Health (MOH), Tourist Board and Public Health workers. It is also worth noting that these key institutions are mandated to implement the HACCP system and educate food service providers and consumers in Ghana.

### Data collection and analysis

Fifty (50) questionnaires were administered in two wards of the Bantama and Oforikrom sub metropolitan areas during the pilot study. These two wards for the pilot study were excluded from the final data collection to minimise spillover effects. The questionnaire was further edited based on the feedback from academics and professionals in the food service. Key issues which were mainly clarity, wording and sequencing of questions were corrected before the final questionnaires were administered. Data on food safety particularly on hazard analysis and critical control point (HACCP) as a food safety policy [34,35] were collected as per the key variables as follows: Information on their background characteristics such as age, sex, education level, and type of service provider were collected from food services providers and staff of institutions. These were done using structured questionnaire. For issues pertaining to policy, some policy documents on adherence to food preparation guidelines were examined with checklist. Information on the policy frame-work on food safety measure was collected from stakeholders such as legal practitioners, staff of Food and Drug Board, Tourist Board staff and Environmental Health Officers who are the law enforcement agencies. Data sources to assess the extent to which the policy embodies the principles of HACCP included methods of food storage, preparation, hygiene practices and sanitation in and around food establishments as enshrined in the HACCP principles. Data on implementation of HACCP as a food safety policy included, implementing bodies, and procedure for implementation and enforcement and sanctions on non adherence to HACCP by food enterprises. These were collected using structured questionnaire.

Data was analyzed into descriptive statistics, summarized and displayed in tables. For continuous variables, the estimates were for difference in means with 95% confidence interval, 5% significance levels. Data entry was done using SPSS version 17. To test the strengths of some selected variables, Odds ratios (OR) with 95% confidence intervals (CI) were calculated using logistic regression, Table 1.

**Table 1:** The strengths of some selected contextual factors on the practice of HACCP

**Variable**	**OR**	**95% CI of OR**
**Educational level**		
High level	Ref	
Low level	2.27	1.16-7.45
**Food service providers**		
Matron	Ref	
Food vendor	0.68	0.12-3.84
Restaurant operator	9.75	1.52-62.63
**Institutions**		
FDB	Ref	
KMA	2.00	0.16-25.12
Tourism board	40.00	3.58-447.03
MOH (Food & nutrition)	5.14	0.45-59.46

### Institutional review board approval

Permission was sought from study population. Verbal as well as written consent for the study were obtained from owners, employees and managers of hotels, restaurants, ‘chop bars’ (traditional food operators), staff from Food and Drugs Board, Tourist Board and Environmental Health Officers. All information collected, remained confidential for the purpose of the research only. The committee for Human Research Publications and Ethics (CHRPE) of Kwame Nkrumah University of Science and Technology (KNUST) provided ethical clearance in line with the Helsinki Declaration for the study.

## Results

### Background characteristics

To understand respondents’ background in relationship to their responses to questions posed to them in this survey, Table 2, they were asked a series of questions on the seven principles of HACCP. A total of four hundred and fifty food service providers were interviewed. Eighty-four (18.7%) were males and three hundred and sixteen (81.3%) were females with the highest age of 55 years; the lowest age of 18, mean 33.1 and standard deviation of 7.5 years. A majority of respondents were 28 years of age with most food service providers attaining basic education (Middle/Junior High School certificate). Slightly over a tenth had attained tertiary level of education with.

**Table 2:** Socio-demographic characteristics of respondents

**Characteristic**	**Frequency n = 450**	**Percent**
**Mean Age (STD)**	33.1 (7.5)	
**Sex**		
Male	87	18.7
Female	366	81.3
**Level of education**		
No formal Education	120	26.7
Middle/Junior High School	174	38.7
Senior High/Tech Education	101	22.4
Diploma	38	8.4
Degree	17	3.8
**Type of practitioner**		
Matron	28	6.2
Food vendor	357	79.3
Restaurant operators	65	14.4
**Full time employee**		
Yes	420	93.3
No	30	6.7
**Channel of food distribution**		
Wholesaler-Retailer-Final consumer	8	1.8
Retail-Final consumer	49	10.9
Final consumer	393	87.3
**Managers by rank**		
Senior	42	84.0
Junior	8	16.0
**Managers by institution**		
EHO	10	20.0
CHNO	10	20.0
PHO	10	20.0
FDBO	10	20.0
GTBO	10	20.0

*Abbreviations*: EHO environmental officer, CHNO community health nutrition officer, PHO public health officer, FDBO Food and Drugs Board Officer, GTBO Ghana Tourist Board Officer.

Out of the four hundred and fifty food service providers, majority 375 (79.3%) were food vendors and 65(14.4%) were restaurant operators. As high as 93.3% of the food service providers were full time workers. In all, fifty stakeholders were interviewed. Out of fifty key stakeholders interviewed, 84% were senior officers who had worked for more than five years while the rest had worked for between two and three years, were junior staff, and from environmental health division, Food and Drug Authority Community Health & Nutrition and Public Health and the Tourist Board, (Table 2).

### Policy on HACCP and its adherence of food preparation guidelines

Table 3 shows that, HACCP as a food safety policy is not adhered to by most food service providers as 375 (83.3%) responded ‘No’ to HACCP being part of food safety guidelines and ‘No’ to HACCP forming the basis of their food safety guidelines. It was revealed that the commonest monitoring procedure used by food service providers was observation. For record keeping, 416 (92.4%) did not follow any guideline to check whether laid down procedures were followed. For those who had some aspects of HACCP, i.e. normal hand washing was not even regularly adhered to. On what they do when they identify a hazard during food preparation, majority, 147 (32.7) said they threw food away while others did nothing or put in corrective measures.

**Table 3:** Implementation of policy on HACCP and its adherence to food preparation guidelines among stakeholders in food service

**Guidelines**	**Response**	**Frequency n = 450**	**Percent**
HACCP principle part of food	Yes	75	16.7
Safety guidelines	No	375	83.3
HACCP form basis of food safety guidelines	Yes	75	16.7
	No	375	83.3
Keep records on procedures	Yes	83	18.4
To ensure safe food	No	367	81.6
Type of monitoring procedures	Check list	39	8.7
	Observation	286	63.6
	Cooking delicious meal	57	12.7
	Comparing how others prepare food	68	15.1
Have a guide to check on laid down procedures.	Yes	34	7.6
	No	416	92.4
Reaction to hazard identified during food preparation	Cook food well	60	13.3
	Throw food away	147	32.7
	Put corrective measure	46	10.2
	Do nothing	5	1.1
	No response	192	42.7

### Managers’ perception on HACCP policy and its adherence in food preparation

From Table 4, it is seen that food safety policies were not fully adhered to by food service providers. In an interview with stakeholders (Law enforcing agencies), it was made clear that only food and drug law (PNDC law 305), and other local assembly bye laws are enforced. It was also evident that only one food factory was in the known to practice HACCP. Thirty-four (68%) said HACCP guidelines were not mandatory while others said it was mandatory. Sixteen (32%) said HACCP as a food safety guideline was difficult to practice or adhere to while majority said it was not difficult to practice. A little over 10% said service providers were aware of HACCP while others said service providers were not aware.

**Table 4:** Perception of managers on policy on HACCP and adherence to food preparation guidelines

**Questions**	**Response**	**Frequency, n = 50**	**Percent**
Are guidelines mandatory?	Yes	34	68.0
	No	16	32.0
Difficult to practice HACCP	Yes	16	32.0
	No	34	68.0
Food service staff aware of HACCP	Yes	6	12.0
	No	44	88.0
Service staff well informed about other guidelines	Yes	12	24.0
	No	38	76.0
	Through education	9	18.0
Preventing food borne diseases	Enforcement of laws	10	20.0
	Creation of public awareness	7	14.0
	Through education and enforcement of laws	4	8.0
	Through education and creation of public awareness	1	2.0
	All of the above	19	38.0

Again about a quarter (24%) said service providers were not well informed on other food safety guidelines while majority said service providers were well informed about safety guidelines. For the prevention of food borne infections, majority said this was done through education, public awareness creation and enforcement of the law, education alone and enforcing food safety regulations only.

### The extent to which the policy embodies the principles of HACCP

Few questions were asked on the extent to which policy embodies the principles of HACCP. Respondents were asked if HACCP formed the basis of food safety policy, and whether stakeholders encouraged the practice, kept records and also checked if laid down procedures were followed in food preparation, (Table 5). Stakeholders who enforced the laws on food safety admitted that food safety policies did not embody HACCP. Fifteen (30.0%) said HACCP principles were part of food safety guidelines while others thought otherwise. Few, 12 (24.0%) said HACCP principles formed the basis of food safety guidelines but most respondents said it did not form part of food safety guidelines with (36%) saying regulatory bodies encouraged food service providers to practice HACCP. For those who said regulatory bodies encouraged the enforcement of HACCP as the basis of food preparation guidelines, 20 (62.5%) said it was because of their level of education while others attributed it to inadequate resources and lack of legal backing. On whether they had guidelines to check whether service providers followed laid down procedures, only 11 (22%) answered yes.

**Table 5:** The extent to which policy embodies the principles of HACCP among managers

**Question**	**Response**	**Frequency, n = 50**	**Percent**
HACCP principles part of food safety guidelines	Yes	15	30.0
	No	35	70.0
HACCP forming the basis of food safety policy	Yes	12	24.0
	No	38	76.0
Encourage practice of HACCP	Yes	18	36.0
	No	32	64.0
If no, why don’t you?	Low level of education	20	62.5
	Inadequate resources	9	28.1
	Lack of legal backing	3	9.4
Records on procedures to ensure safe food	Yes	14	28.0
	No	16	72.0
Guidelines to check procedures	Yes	11	22.0
	No	39	78.0

### The implementation of the policy in the context of HACCP

One of the preliminary questions put to food service providers was whether they knew of any institutions that enforced the laws on food safety, organized educational programme on HACCP when they visited their premises and as to whether they, the food service providers, had the license to operate. Questions were also put to stakeholders to determine the monitoring and implementation of HACCP, its effectiveness and impediments if any, (Table 6).

**Table 6:** Implementation of policy in the context of HACCP stakeholders in food service

**Question**	**Response**	**Frequency**	**Percent**
Knowledge of institution or body	Yes	297	66
Which enforces law on food safety	No	153	34
Visit to food premises on regular basis	Yes	168	37.3
	No	282	62.7
Education on HACCP	Yes	27	16
	No	141	84
Number of visit	Once a year	112	24.9
	Once a week	83	18.4
	Once in three months	233	51.8
	Twice a week	22	4.9
Having license to operate food	Yes	195	43.3
Establishment	No	255	56.7
If no why?	It is not necessary	65	25.5
	It is not compulsory	174	68.2
	It does not serve any purpose	16	6.3
Period of renewal of license	Every six months	30	15.4
	Every year	147	75.4
	Every two years	13	6.7
	Every three years	5	2.5

With regards to HACCP implementation, (Table 6), 297 (66%) knew institutions responsible for enforcing food safety, 153 (34%) said they did not know the existence of such bodies, 168 (37.3%) said such bodies visited their premises and 282 (62.7%), said they were never visited, 423 (94%) said they did not receive any form of education from such bodies. For those who had received some education, it was on renewal of medical certificate and issuance of court summons. When asked whether service providers had medical certificate/license, 195 (43%) said yes while the rest responded no.

In (Table 6), regarding why food service providers did not have medical certificate, majority 174 (68.2%) said it was not compulsory and others answered it did not serve any purpose or not necessary. For those who had medical certificate, they renewed it every six months, yearly, every two years, and every three years. All key stakeholders 50 (100%) answered no to implementation and certification of HACCP policy. Key stakeholders in an interview admitted that there was no implementation and certification of HACCP because it had not being accepted as a policy in Ghana. When respondents were asked what they did when they identified a hazard during food preparation, 60 (13.3%) said they cooked food well, 147 (32.7%) said they threw food away, 46 (10.2%) said they put in corrective measures, and 5 (1.1%) did nothing and 192 (42.7%) declined to answer.

### Implementation of policies in the context of HACCP among managers

Table 7 shows the implementation of guidelines in the context of HACCP by stakeholders. For the 50 stakeholders, 25 (50%) said their organizations considered HACCP as food safety measure which needs to be implemented. On how HACCP policy could be implemented, 25 (50%) said through education and training while others mentioned law enforcement and integrating it into other food safety measures. On the other hand, 12 (24%) said none of these and 10 (20%) said none of the first two. Fourteen (28%) said their organizations conducted training on HACCP for food service providers but the rest said their organizations did not. Regarding how stakeholders monitored the implementation of HACCP, 15 (30%) did it through regular inspection, 17 (34%) used observation and others did no monitoring. On how often stakeholders visited food service providers premises, majority visited once a week or once a while. Again, 43 (86%) considered HACCP a good food safety policy and 7 (14%) did not consider it as a good food safety policy. Also, because HACCP did not form part of food safety policies, stakeholders (Law enforcing agencies) were unable to implement it.

**Table 7:** Implementation of policies in the context of HACCP among managers

		**Frequency, n = 50**	**Percent**
HACCP considered as a food safety measure	Yes	25	50.0
	No	25	50.0
Strategies used to Implementing HACCP	Through law enforcement	1	2.0
	Through education/training	25	50.0
	Integrating with safety guidelines	2	4.0
	None of the above	12	24.0
	Law enforcement	10	20.0
Conduct training for food staff	Yes	14	28.0
	No	36	72.0
Number of times inspection is done	Twice a week	3	6.0
	Once a month	8	16.0
	Once a week	5	10.0
	Once a while	34	68.0
Method of Monitoring HACCP	Observation	17	34.0
	Through research process	1	2.0
	No monitoring is done	17	34.0
HACCP considered a good food safety policy	Yes	43	86.0
	No	7	14.0
Effectiveness of monitoring	Very effective	7	14.0
HACCP implementation	Not effective at all	37	74.0
	Somehow effective	2	4.0
	None of the above	4	8.0
Factors impeding effectiveness	Inadequate logistics	31	62.0
	Lack of knowledge on HACCP	17	34.0
	Political interference	2	4.0
	Lack of planning	0	0.0

### The strengths of some selected contextual factors on the practice of HACCP

The study further explored the strengths of relationship between some selected study variables, (service providers’ education, type of provider, and institutions), (Table 1), on the practice or adherence to HACCP and food guidelines. Providers with higher education were twice likely to adhere to HACCP and food guidelines. With reference to matrons, food vendors (informal food sellers) were 68% less likely, (OR = 0.68, 95% CI = 0.12-3.8) while restaurant operators were 10 times more likely, (OR = 10, 95% CI = 1.5-62), to practice HACCP. In terms of Food and Drug Board, the Ghana Tourist Board recorded the highest Odds of ensuring that food operators adhere to HACCP and food guidelines, (OR = 40, 95% CI = 35.8-44.7) than Public Health officers, (OR = 5, 95% CI = 0.45-59) and KMA, (OR = 2, 95% CI = 0.16-25).

## Discussion

Food is a source of energy and vitality for man but food is also the sources of infection for many of the communicable diseases. Presently, it appears that prepared food are not tested neither do a majority of people involved in the preparation and sale undergo medical screening. In spite of the existing regulatory bodies such as the Food and Drug Board, Ghana Tourist Board, Metropolitan Assemblies, none of these bodies is actively involved in ensuring that prepared food sold to the general public is wholesome. Though food screening and inspection are done, the ultimate regulation is Hazard Analysis and Critical Control Point which is known and accepted worldwide as one of the effective food safety tool. A cross sectional study of 450 food operators and 50 managers from 5 regulatory bodies was undertaken to assess the existing situation regarding implementation of HACCP and adherence to food safety guidelines.

### Background characteristics

The results revealed that the mean age of respondents was 33.1 years with a standard deviation of 7.5 years. The highest age was recorded to be 55 years; the lowest age was 18 years. A majority of respondents were 28 years of age (10.2%) and most food service providers were females and also in full time business. Only a few had obtained higher level of education while a majority had low level of education. Out of the fifty key stakeholders, forty-two (84%) were senior officers who had worked for more than five years. The low level of education of respondents could be a contributing factor to the non-adherence to HACCP, corroborating Italio “and others” [28] and Yapp and Fairman [4], who identified similar situation in research conducted on HACCP and food hygiene in hospitals and small and medium-sized enterprises respectively.

Of the 290 food services staff interviewed, 78.8% were aware of the five leading food borne-pathogens; this knowledge was significantly higher among those with higher educational level and those who worked in hospitals that implemented the HACCP system than those who had lower level of education. Younger staff and those who had attended continuing educational courses on food hygiene and hospital food borne diseases had a significantly higher knowledge of safe temperatures for food storage than older staff who did not have access to continuing education. The number of food services providers and employees reported to be certified was lower, indicating a need to conduct basic food safety training and certification for all levels of food service providers and employees [1]. A majority of key stakeholders interviewed were senior officers who were certified, a number similar to the 71% of food service directors who had food safety certification in a study by Giampaoli et al. [34]. Knowing that these people had a high level of education and work experience, it would be expected to recognize and assess the critical factors of effective implementation of the HACCP system.

### Policy on HACCP and its adherence of food preparation guidelines

The study established that food service providers are likely to adhere to HACCP when they anticipate that the adoption would enhance economic competitiveness such as cost reduction, quality improvement and sales increase as previously revealed by [1,4,9]. In addition, food service providers are also likely to adopt and adhere to HACCP and food safety management systems to conform to food safety issues in order to gain legitimacy such as reputation and recognition [25]. The findings from the study are consistent with a previous study by Oliver [27], where; some institutions adopted HACCP and other food safety management systems because their policies of food safety enabled them to access foreign markets, as a result of large customers demanding their suppliers were “registered”, and also from the demands of the export market. Again, it was realized that only one food establishment was found to practice HACCP in the Kumasi Metropolis.

This presupposes that food establishments which failed to adhere to HACCP principles were not liable to any form of punishment and also the benefits of improvement in food safety have not been realized [12]. Ackah [29] stated that when the law is passed, a competition Commission should be set up to regulate the Ghanaian Market and to ensure access to affordable and quality products and services. Yet there was no evidence of enforcement of the HACCP in food manufacturing. Spearing and others [9], added that core responsibilities should include the establishment of regulatory measures, monitoring system performance, facilitating continuous improvement, and providing overall policy guidance.

The study also revealed that institutions mandated to ensure adherence to HACCP had performed abysmally, to the extent of top management support and commitment had not been up to task (84%). As earlier study revealed, the stronger the belief in HACCP and its importance by top management, the more likely that it would be integrated into the decision-making process [11,13].

### The extent to which food policy embodies the principles of HACCP

This study revealed that no clear policy has been formulated on HACCP in Ghana. The few policies on food safety were found to be the Food and Drugs Law [30], Criminal code Act 29/1960, section 286 and sections of the Metropolitan and District Assemblies Bye Laws that prohibit the sale of unwholesome food to the public. None of these food safety regulations in use considers aspect of HACCP as a means of preventing food infection, though HACCP is designed for use in all segments of the food industry from growing, harvesting, processing manufacturing, distributing and merchandising to preparing food for consumption [8,9,23,30]. No attention had been given to the principle as a system which is explicit and able to detect and eliminate any hazard associated with food preparation and processing [7]. This generally supports the contention that food service staff do not respond uniformly to institutional pressures, but rather adopt varying strategies that depend on the nature of the institutional pressures forced on them. Moreover, the degree of conformity is a strategic choice, which depends on the nature of the pressures, as well as on organizational interests in maintaining legitimacy, support, and economic viability [12,20]. The perceived importance of external stakeholders (i.e. government agencies, the community, food safety organizations and the media) is significantly related to the level of food safety policies. In emphasis, modern food laws should not only contain the necessary legal powers and prescriptions to ensure food safety, but also allow the competent food and other authorities to build preventive approaches into the system [34,35].

### The implementation of the policy in the context of HACCP

The study showed that a majority of stakeholders and food service providers did not implement HACCP. This could be attributed to a number of reasons. First, Panisello and others [26], and Taylor and Kane [33], explained that factors that could influence the adoption of practices to enhance firm’s food safety status and implementation of HACCP seem far more complicated than imagined. They cannot be solely explained in terms of unwillingness by manufacturers but rather by the presence of several technical barriers that may impede the benefits of the application of the HACCP system, since firm level benefits that are attributable to HACCP implementation are intangible [12]. In this study, training was identified as a top barrier, which is consistent with what has been found in the literature [14,19]. Most significant barrier was the lack of opportunities for in-house employee training and outside training. The human resource attributes that were significant factors influencing HACCP effectiveness were also mentioned in the study of Vela and Fernandez [19].

Time, money, and other resources were also cited by stakeholders as being management barriers, employee barriers identified included time and training [16,18,34]. Food services staff also indicated that they could implement HACCP effectively if they were provided with sufficient guidance and support in a context of general consensus of the HACCP terminology and requirements.

Also, as established elsewhere, inadequate analysis of food-borne safety hazards and implementation in the food sector resulted from the lack of understanding by employees as well as problem of attitudinal level of employees [33]. In exploring experiences, the small proportion of the food service staffs that implemented HACCP irrespective of the level of effectiveness they achieve means that in implementing such a system, a number of benefits are derived on behalf of a company, either internal or external ones, such as protecting the business from otherwise unforeseen problems and providing evidence of “due diligence” [22]. The role of governments and professional trade bodies in facilitating HACCP implementation and devising strategies to overcome barriers that are faced by Small Medium Enterprises (SMEs) were highlighted [10,11]. Predictors of implementation of HCCP policy and adherence with food preparation guidelines were identified as education, type of food operator and type of regulatory body.

## Conclusions

The study found that most food service providers were females, had basic formal education and were not aware of hazard analysis and critical control point (HACCP) as part of food safety guidelines. Staff of food regulatory agencies had mixed feelings but were more inclined to the notion that HACCP principles are not mandatory and do not form the basis of food safety guideline as there is no guideline to ensure that the principles of HACCP are adhered to in Ghana. Though HACCP forms the basis of food safety guidelines globally, the study found that existing food safety guidelines in Ghana do not incorporate the principles of HACCP. Further study to look into feasibility, acceptability and implementation of HACCP in Ghana could be helpful.

### Practical implications

The conclusions suggest that food produced in Ghana is likely to fall short of international standards and may not be unwholesome. Consequently, food borne diseases and related mortality are eminent, this may further increase the huge economic and public health burden on the economy of Ghana and other national economies and retard the realization of the millennium development goals.

### Limitations of the study

The major limitation of the study could be respondents’ bias. It was possible that respondents’ were influenced by social desirability. However, this was triangulated by extensive literature review and interviews with both regulatory bodies and food service providers. Again, assessing adherence to implementation guidelines in a snap short may not reveal the true situation as adherence is a behavioural variable and needs to be studied within a time lag.

### Future research

Further cross sectional studies are needed across Ghana and beyond to assess the current situation. Cohort and experimental studies are needed to follow up both implementers of regulations and food service operators to elicit predictors and the extent of compliance to HACCP policy and adherence to food safety guidelines. This study only explored the existing situation which is needed for the design of a long term study.

## Abbreviations

HACCP: Hazard analysis and critical control point; CGMPS: Current good manufacturing practices; CCP: Critical control points; MDAs: Ministries, departments and agencies; CHRPE: Committee for human research publications and ethics; KNUST: Kwame Nkrumah University of science and technology; GTB: Ghana tourist board; GSB: Ghana standard board; KMA: Kumasi metropolitan assembly; FDB: Food and drugs board; MOH: Ministry of health; SME: Small medium enterprises; TB: Tuberculosis.

## Competing interests

The authors declare that they have no competing interests.

## Authors’ contributions

The authors PA-B, KBS and EAA conceived and implemented the study. KBS supervised data collection from the field. The analysis and interpretation of results were performed by PA-B, KBS and EAA. PA-B led and coordinated the final report writing and developed the first draft of the manuscript. All authors read and approved the final manuscript.
